# Maintaining genetic integrity of coexisting wild and domestic populations: Genetic differentiation between wild and domestic *Rangifer* with long traditions of intentional interbreeding

**DOI:** 10.1002/ece3.3230

**Published:** 2017-07-26

**Authors:** David G. Anderson, Kjersti S. Kvie, Vladimir N. Davydov, Knut H. Røed

**Affiliations:** ^1^ Department of Anthropology University of Aberdeen Aberdeen UK; ^2^ Department of Environmental Studies University College of Southeast Norway Bø in Telemark Norway; ^3^ Department of Basic Sciences and Aquatic Medicine Norwegian University of Life Sciences Oslo Norway; ^4^ Peter the Great Museum of Anthropology and Ethnography (Kunstkamera) Russian Academy of Sciences St. Petersburg Russia

**Keywords:** domestication, Evenki, indigenous animal husbandry, interbreeding, introgression, male‐mediated gene flow, reindeer husbandry, reproductive isolation, Russia

## Abstract

This study investigates the genetic effect of an indigenous tradition of deliberate and controlled interbreeding between wild and domestic *Rangifer*. The results are interpreted in the context of conservation concerns and debates on the origin of domestic animals. The study is located in Northeastern Zabaĭkal'e, Russia at approximately 57 degrees North latitude. Blood and skin samples, collected from wild and domestic *Rangifer*, are analyzed for their mtDNA and microsatellite signatures. Local husbandry traditions are documented ethnographically. The genetic data are analyzed with special reference to indigenous understandings of the distinctions between local domestic types and wild *Rangifer*. The genetic results demonstrate a strong differentiation between wild and domestic populations. Notably low levels of mtDNA haplotype sharing between wild and domestic reindeer, suggest mainly male‐mediated gene flow between the two gene pools. The nuclear microsatellite results also point to distinct differences between regional domestic clusters. Our results indicate that the Evenki herders have an effective breeding technique which, while mixing pedigrees in the short term, guards against wholesale introgression between wild and domestic populations over the long term. They support a model of domestication where wild males and domestic females are selectively interbred, without hybridizing the two populations. Our conclusions inform a debate on the origins of domestication by documenting a situation where both wild and domestic types are in constant interaction. The study further informs a debate in conservation biology by demonstrating that certain types of controlled introgression between wild and domestic types need not reduce genetic diversity.

## 1 INTRODUCTION

Nature conservation aims to maintain biological diversity, which is often defined by the highest level of genetic diversity thought necessary to ensure the evolutionary potential for any species (Slatkin, 1987; Frankham, 2010). Genetic introgression from non‐native, translocated representatives of the same species, or from domesticated versions of a species, is often represented as a key concern or even a threat in the conservation literature, as it may reduce fitness through the introduction of maladaptive traits or by disrupting positive epistatic interactions (Abbott, 2013; Mager, 2013; Wayne & Shaffer, 2016). On the other hand, bioarchaeologists often assume that domestic animals are descended from wild prototypes, and thereby imply that gene flow from wild to tame is necessary to maintain the fitness of the domestic type (Vigne, 2011; Zeder, 2012). However, little is known about the techniques that herders use to manage domestic populations in places where wild populations may pose a risk, or a benefit, to the local herds. Understanding such management techniques would be particularly interesting, as they would necessarily influence the genetic integrity of both domesticated and wild populations. Traditionally, biologists have assumed that herdsmen enforced absolute reproductive isolation between wild and domestic forms (Price, 1984; Driscoll, 2009). This strategy would also maintain the genetic integrity of wild populations unintentionally. However, new models of domestication suggest that while spatial separation of wild and domestic types is often sought, various domestic species ranging from pigs to horses have also been cross‐bred for specific characteristics by allowing controlled introgression from wild herds. (Ottoni, 2013; Jónsson, 2014; Frantz, 2015). For most domestic breeds, this idea remains a hypothesis as the original free‐ranging wild forms have become largely extinct or restricted to a few isolated areas (Clutton‐Brock, 1987). The exact husbandry techniques, which may have been employed in the Neolithic, can only be guessed at. However, this is not the case for reindeer.

Reindeer, *Rangifer tarandus*, are a species commonly considered to be in an early phase of domestication and which often coexist with wild forms (Baskin, 2000; Reimers & Colman, 2009). Today, almost 50% of the approximate 3,000,000 reindeer in the Old World are wild animals, and wild and domestic herds are managed in close coexistence in many areas of Eurasia and in Alaska (Syroechkovskii, 1995; Baskin, 2005). Therefore, this species provides a rare opportunity to link techniques of interbreeding of domestic herds and their wild relatives to their genetic signatures. Despite their value as a proxy for studying the history of domestication, most existing theories of the origin of reindeer husbandry have not approached this problem genetically. The most authoritative theories are cultural historical models which survey the geographic diffusion of different styles of pastoralism (Wiklund, 1918; Maksimov, 1928; Vasilevich & Levin, 1951). They tend to focus on whether or not varying husbandry techniques could have been independently invented, or conversely, if they could have been simply copied and applied to local populations of wild *Rangifer*. They tend to assume that any *Rangifer*—wild or tame—can be harnessed given enough patience and if one had the right equipment. The question of whether domestic reindeer are genetically distinct has never been tested.

Varying degrees of gene flow between wild and domestic reindeer have been reported in several recent studies (Mager, 2013; Colson, 2014; Røed, 2014). These studies primarily focus on degree of introgression of domestic lineages into the native wild gene pool, and which population and environmental factors may affect this. However, the complementary side to this model—the introgression of wild genes into domestic lineages—has been rarely studied. This process may in fact shed more light on the impact of introgression on species diversity.

Northeastern Zaibaĭkal'e constitutes an area of approximately 132,000 square kilometers defined by the Stanovoe mountain range. It is characterized by an alteration of alpine peaks of 1,200 m with year‐round snow packs, interspersed with high open meadow plateaus (500–100 m) and stretches of taiga. Both wild and domestic reindeer herds coexist today in the region. It is likely that this coexistence has persisted for a long time. Zabaĭkal'e has been suggested to be one possible origin point for reindeer husbandry (Wiklund, 1918; Maksimov, 1928; Pomishin, 1990), with Okladnikov and Mazin (1976) speculating that its roots go back to the second millennium BCE. Furthermore, the region is particularly interesting because of its specific herding techniques. It has been documented that local Evenki herdsmen maintain a traditional practice of selectively cross‐breeding domestic female *Rangifer* with wild males to produce offspring named locally as *bai͡unchikan* [*bai͡unchikar ‐*pl], which are often valued as transport reindeer (Shirokogoroff, 1929; Vodop'i͡anov, 1970b; Davydov, 2014). Local herdsmen claim that controlled intermixture with wild populations improves the resilience and strength of the domestic herd. One influential cultural–historical study used this practice of interbreeding as a criterion for distinguishing the style of reindeer husbandry in Zabaĭkal'e from that of neighboring regions (Vasilevich, 1964). Evenki reindeer husbandry in Zabaĭkal'e thereby presents itself as a unique case for studying how introgression of wild alleles into the domestic gene pool may have been managed during the early processes of domestication. To shed light on the potential for introgression and relevant circumstances, we combined ethnographic and genetic methods.

In this article, we test the hypothesis that Zabaĭkal Evenkis are able to enforce a strict genetic separation of wild and tame *Rangifer,* despite local breeding practices that tolerate or even to encourage controlled inter‐breeding. We hypothesize further that their use of strict criteria to control genetic introgression through culling or castration is key to maintaining this divide. A strong genetic difference between the wild and domestic populations would therefore document their pastoral skill in maintaining and controlling specific reindeer pedigrees. Establishing the identity and level of distinctiveness of wild and domestic herds is further important to settle an old debate on whether or not a domesticated harness reindeer constitutes a distinct type of reindeer, compared to wild reindeer.

## 2 MATERIAL AND METHODS

### 2.1 Study populations

**Figure 1:**
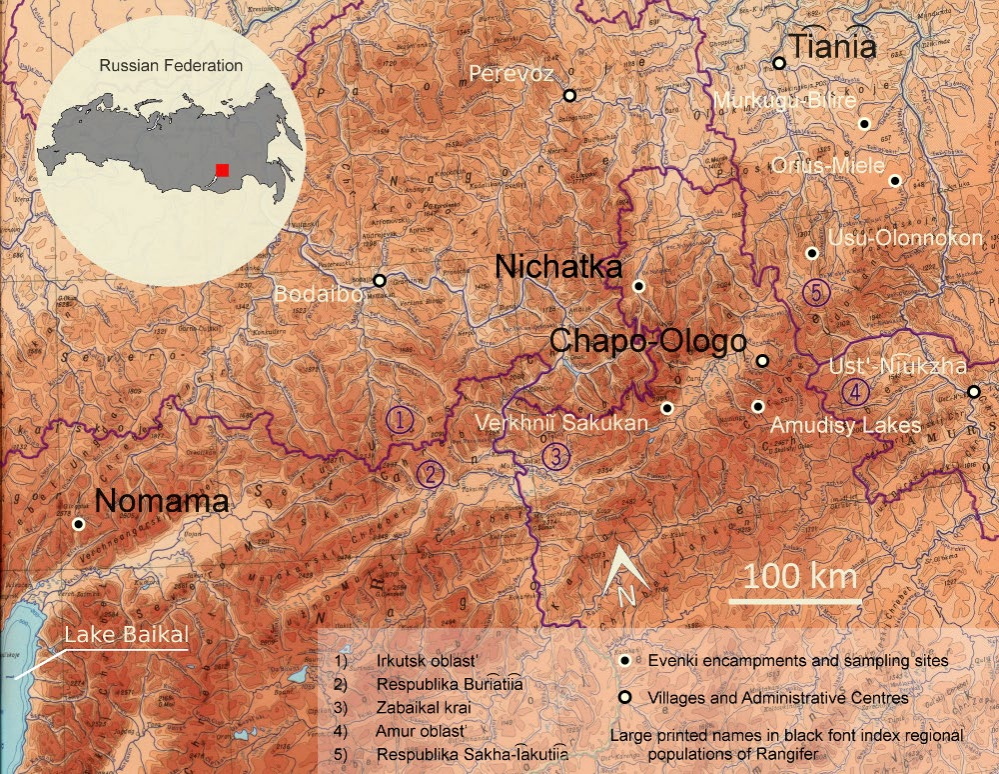
The locations of the local encampments and sampling sites, as well as regional populations, for wild and domestic *Rangifer* in northeastern Zabaĭkal'e. The boundaries of the local political districts, which bisect the region, are indicated with numbers

The reindeer in this study were sampled between 2012 and 2014 from wild and domestic regional populations around the settlements of Chapo‐Ologo and Ti͡ani͡a and the encampment at Nomama. We have included also data from a previous study of an Evenki herd at Lake Nichatka sampled in 2001 and 2002 (Røv & Abe, 2002; Røed, 2008). The name of each population corresponds to the residential base for the constellation of families holding local herds around that site and hunt wild reindeer in that same region (Figure 1). During the Soviet period, some of these residential bases would have been the headquarters for one centrally organized state farm, which exercised a great influence on the reproduction of each regional domestic population. We understand a regional domestic population to be a larger grouping of animals, which for historic and geographic reasons probably have been in constant interchange.

It is difficult to estimate the total number of wild and domestic *Rangifer* as this cultural historic region crosses four political districts and official statistics are fragmentary (Table A1). Existing government statistics record approximately 7,000 domestic reindeer in the region (although local informants claim that number is greatly underestimated) (Federal'nai͡a Sluzhba Statistiki, 2016). A different set of accounts list approximately 24,000 head of wild reindeer (although that figure is said to be exaggerated) (Pavlov, 2016). In every context where we spoke with local herders, we were told that wild reindeer were rare. Wild reindeer were listed as an endangered species in Buri͡atii͡a until 2005 (Boĭkov, 2005).

The Chapo‐Ologo regional domestic population consists of samples obtained from local herds with biographies suggesting they were offspring of the original Charskiĭ state farm, which in turn combined the smaller herds of dozens of indigenous families living in the region before collectivization. The domestic data set represents herds, or portions of herds, kept by four extended families. The set of wild reindeer associated with the Chapo‐Ologo domestic population was hunted primarily in the Verkhniĭ Sakukan valley with a few samples from the Amudisy Lakes. The Ti͡ani͡a region captures the territory of two clan communities Ti͡ani͡a and Tokko which have restored reindeer husbandry after it nearly disappeared in 1993 when the local state farm was disbanded. The contemporary domestic regional population was assembled from a small population of a few dozen local domestic reindeer with influxes of five to fifty reindeer from Lake Nichatka, Chapo‐Ologo, Perevoz, and Ust’‐Ni͡uzhka and another five regional encampments between 1993 and 2010. Domestic animals born in this territory were grouped together as the Ti͡ani͡a regional population. It represents the herds or portions of herds kept by five extended families. The wild reindeer samples of this region were from animals hunted on the Ori͡us‐Milele and Usu Rivers. The domestic Nomama samples were from domestic reindeer held by the clan community corporation “Ulutki”—essentially one extended family—which is headquartered at the ice‐covered headwaters of the Lena River. This region was once the home to the state farm “Severnyĭ” that was disbanded in 1976. The region was left empty of domestic reindeer for 15 years, albeit with a large population of feral reindeer. Domestic reindeer husbandry was reintroduced in the region first in 1992–1993, according to informants by first lassoing the local feral reindeer, and more substantially by the purchase of thirty head of reindeer from the Chapo‐Ologo region. The wild reindeer hunted in the Nomama region is likely separated from all the other wild herds by significant mountain ranges over large geographic distances (more than 500 km). In contrast to all the other sites, the extended family at Lake Nichatka never had participated in collectivization. Their kinship networks for the most part looked northwards the Bodaĭbo district of Irkutsk *oblast’* (Anderson et al., 2014) which facilitated the exchange small groups of 5–10 head of reindeer. The wild reindeer from this setting were hunted within 25 km of the main residential camp and could be considered to be part of a wild population which moves freely between this site and sites across the border to Ti͡ani͡a region.

### 2.2 Rangifer life histories

For this study, we collected detailed pedigrees and descriptions of wild and domestic *Rangifer* from local herdsmen at Chapo‐Ologo, Tia͡nia͡, and Nomama. A total of 117 domestic animals were documented of which 101 were used in this study. Fifty‐six wild *Rangifer* were described of which 49 samples were used here. The descriptions of domestic animals were structured around a set of open‐ended questions eliciting the life history of a particular animal. The interviews, at minimum, affixed the age, sex, place of birth, and ownership status of each animal. Beyond this we photographed each animal and recorded detailed observations of the reindeer type, its pelage, its role in the social organization of the herd, peculiarities of its behavior, and how it might have been harnessed or tamed for other activities. The latter information was important for eliciting what qualities the herders valued in a domestic animal. We recorded information in the Russian, I͡Akut, and Evenki languages in order to capture details which may translate poorly. Information on wild *Rangifer* was confined to visual assessments of the age, sex, and qualities of the hunted wild animal.

The previously published set from Lake Nichatka are documented only by sex and type (wild/domestic) with some specific biographical information on individual animals available in Abe (2005). Of the 35 domestic and 15 wild samples originally gathered, 31 domestic and 13 wild samples were used in this study. In 2012, we interviewed one Evenki herder who worked with this local herd for further details on this regional population.

### 2.3 DNA extraction

DNA was obtained from hair follicles, skin samples, or from FTA cards designed for forensic work (Smith & Burgoyne, 2004). The tissue and blood samples were all noninvasive taken from animals culled, or ear‐clipped, during normal pastoral activities. DNA extraction of skin samples was performed using DNaeasy Blood & Tissue Kit (Qiagen) following the manufactures protocol. DNA from hair follicles was extracted using the chelex method (Walsh, 1991). DNA from FTA cards was extracted from a 0.4 × 0.4 cm piece of the FTA cards using the boiling method, as described in Kvie (2016).

### 2.4 Mitochondrial DNA analyses

A 503‐base pair (bp) long fragment from the mitochondrial control region (CR) was amplified using the forward primer RtCRF (5′‐AAT AGC CCC ACT ATG AGC ACCC‐3′) (Flagstad & Røed, 2003) and the reverse primer RtCR‐528 (5′‐TAG GTG AGA TGG CCC TGA AGA AA‐3′) (Bjørnstad & Røed, 2010). Amplification was performed using the following program: 95°C for 2 min, 95°C for 30 s, 55°C for 30 s, and 72°C for 1 min (step 2–4 cycled 30 times) and finally, 72°C for 10 min. PCR reactions were performed in 20 μl total volume using 1–2 μl DNA template, and with the following final concentrations: 1× buffer, 1.5 mmol/L MgCl_2_, 0.8 mmol/L dNTPs, 5 pmol of each primer, 0.5 μg/μl Bovine Serum Albumin (BSA), 0.5 U/μl AmpliTaq DNA polymerase (Applied Biosystems), and dH_2_O to make up the remaining volume. The samples were cleaned for unincorporated primers and nucleotides using Illustra ExoProStar (GE Healthcare) diluted 10 times. Cycle sequencing was performed in a 10 μl reaction volume, using BigDye v3.1 sequencing kit (Applied Biosystems) following the manufacturer's recommendations. Purification was carried out using standard EDTA/EtOH precipitation. Capillary electrophoresis and data analysis was performed with an ABI 3130xL‐ or 3500xL instrument (Applied Biosystems). All sequences were sequenced in both directions, and the consensus sequences were aligned by ClustalW (Thompson, 1994) and edited in MEGA5 (Tamura et al., 2011).

### 2.5 Microsatellite analyses

All samples were analyzed for 13 reindeer‐specific microsatellites (NVHRT‐01, NVHRT‐03, NVHRT‐16, NVHRT‐21, NVHRT‐31, NVHRT‐48, NVHRT‐73, NVHRT‐76 (Røed & Midthjell, 1998), RT‐1, RT‐5, RT‐6, RT‐9, RT‐27 (Wilson, 1997). The amplification was performed on a GeneAmp PCR System 9700 (Applied Biosystems) as previously described (see methods described in Røed (2002)). PCR products were electrophoresed using an ABI Prism 3500xl Genetic Analyzer (Applied Biosystems). Use of these markers in a previous study has given evidence of low scoring errors (<5%) due to stutter bands, allelic dropout, or null alleles (Røed, 2008).

### 2.6 Statistical genetic analyses

CR polymorphism estimates in terms of number of haplotypes (Nh), gene diversity, and nucleotide diversity were calculated in DnaSP (Librado & Rozas, 2009). CR genealogical relationships were examined by constructing a median‐joining network (Bandelt, 1999) using Network v4.6 (Fluxus Engineering, 2016).

For both the CR and microsatellite data, we used Arlequin v.3.5 (Excoffier & Lischer, 2010) to calculate pairwise genetic distances (*F*_ST_) to examine genetic differentiation among populations. We also used Arlequin to perform analysis of molecular variance (AMOVA) (Excoffier, 1992) to estimate the proportion of genetic variation found among populations (*F*_ST_), among populations within groups (*F*_SC_), and among groups (*F*_CT_). The AMOVA was run with all samples, imposing no hierarchical structure, and with the populations divided into two groups, separating between domestic and wild populations. Significance associated with the fixation indexes was evaluated through random permutation procedure using 10,000 permutations.

We used GenALEx v.6.5 (Peakall & Smouse, 2012) to calculate microsatellite genetic diversity in terms of number of different alleles (Na), number of effective alleles (Ne), observed heterozygosity (Hobs), expected heterozygosity (Hexp), and number of private alleles. Bayesian assignment, as implemented in the program STRUCTURE 2.3.4 (Pritchard, 2000), was used to assess whether discontinuities existed in the distribution of genetic variation within the data set. For each number of genetic clusters (*K *∈ [1,8]), a model with uniform priors, admixture, correlated allele frequencies, 20,000 burn‐ins, and 200,000 Markov Chain Monte Carlo (MCMC) iterations was run ten times. For each *K*‐value, average posterior probability among runs and standard deviation (SD) was calculated. Clumpp (Jakobsson & Rosenberg, 2007) was used to choose the most representative of the ten runs. Data were graphed using STRUCTURE PLOT (Ramasamy, 2014). To identify the proportion of individuals with mainly domestic, wild, or mixed ancestry, we used the STRUCTURE analysis but with only two inferred populations, namely domestic and wild. A threshold posterior probability value of 0.2 < *q* < 0.8 was used to indicate individuals with admixed ancestry.

## 3 RESULTS

### 3.1 Genetic analyses

**Figure 2:**
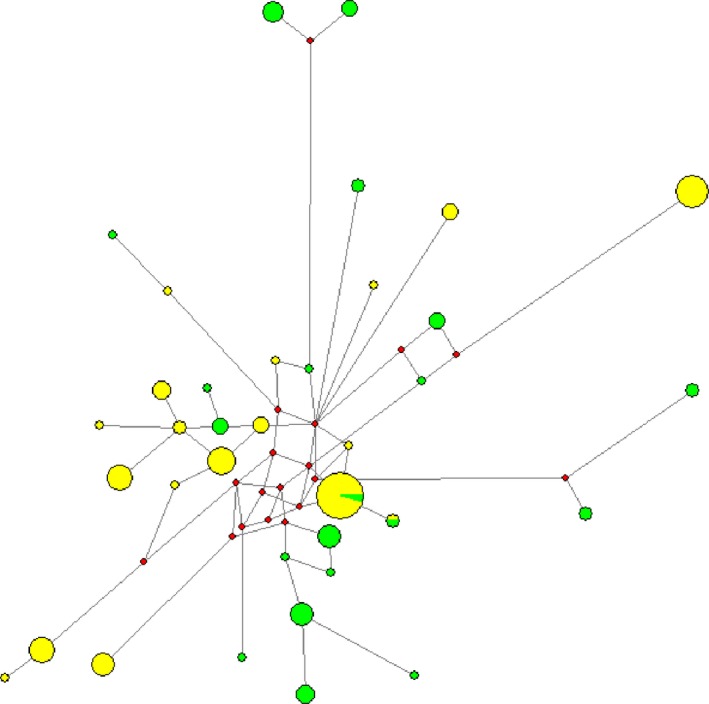
Median‐joining network of mtDNA haplotypes in wild and domestic *Rangifer* in northeastern Zabaĭkal'e. Each circle represents unique haplotypes with area proportional to the number of reindeer sharing a haplotype, and with the “status” (wild or domestic) each color coded with wild in green and domestic in yellow

We identified 18 unique CR haplotypes among the domestic populations and 20 unique CR haplotypes among the wild populations (Table 1). However, only two haplotypes are shared between the two gene pools, with two wild individuals, both from Ti͡ani͡a, having shared haplotype with local domestic reindeer (Figure 2). Also, the *F*_ST_ estimate for genetic differentiation between pooled wild and domestic was significant in the CR (*F*_ST_ = 0.832 *p* < .0001) as well as in the microsatellites (*F*_ST_ = 0.032, 0.010 < *p* < .05). Our results show a higher significance level in the mtDNA dataset compared to the microsatellites. This may be explained by the clonal/maternal mode of inheritance of mtDNA resulting in an effective population size that is one‐fourth of nuclear DNA. Smaller population size makes mtDNA more sensitive to population changes as it is more exposed to genetic drift (Birky, 1983).

**Table 1:** Amount of genetic variation in the microsatellite loci and in the mitochondrial CR in wild and domestic reindeer herds from northeastern Zabaĭkal'e. *N* = number of individuals. For microsatellites, Na gives number of different alleles, and *H*_obs_ and *H*_exp_ gives observed and expected heterozygosity. For the CR Nh gives number of different haplotypes

Status	Code	Location	Microsatellites				MtDNA			
			*N*	Na	*H*_obs_	*H*_exp_	*N*	Nh	Haplotype diversity	Nucleotide diversity
Domestic	1	Nomama	10	5.54	0.652 (0.145)	0.725 (0.092)	10	3	0.38 (0.18)	0.004 (0.003)
	2	Lake Nichatka	31	6.69	0.669 (0.131)	0.744 (0.073)	18	10	0.87 (0.06)	0.012 (0.007)
	3	Chapo‐Ologo	37	7.46	0.707 (0.109)	0.748 (0.053)	36	9	0.84 (0.04)	0.016 (0.009)
	4	Tia͡nia͡	23	6.77	0.701 (0.134)	0.774 (0.082)	24	11	0.85 (0.06)	0.013 (0.007)
	Domestic pooled		101	9.62	0.680 (0.062)	0.767 (0.062)	88	18	0.87 (0.02)	0.014 (0.008)
Wild	5	Nomama	24	8.08	0.736 (0.095)	0.808 (0.071)	24	10	0.91 (0.03)	0.020 (0.010)
	6	Lake Nichatka	13	6.39	0.724 (0.168)	0.760 (0.131)	10	6	0.78 (0.14)	0.009 (0.006)
	7	Tia͡nia͡	7	5.77	0.700 (0.226)	0.804 (0.108)	7	4	0.72 (0.18)	0.007 (0.005)
	8	Chapo‐Ologo	5	4.07	0.636 (0.180)	0.725 (0.128)	5	3	0.80 (0.16)	0.012 (0.008)
	Wild pooled		49	9.69	0.717 (0.064)	0.815 (0.070)	46	20	0.94 (0.02)	0.017 (0.009)

High levels of pairwise genetic differences among the eight regional populations of wild and domestic *Rangifer*, respectively, was evident from the microsatellite markers (Table 2). Significant differences were found among all populations (*p* < .05), except between the regional domestic herd from Ti͡ani͡a and the domestic herds from Nomama and Nichatka (*p* = .06), and between the wild herd from Chapo‐Ologo and the wild herds from Lake Nichatka (*p* = .09) and Ti͡ani͡a (*p* = .208). Pairwise genetic differentiation calculated from the CR data show no or low levels of significance, except for the regional population of Nichatka wild reindeer which is significantly different from all other populations (*p* < .001) (Table 3). For the CR, these tests are not very informative due to relatively low sample sizes obtained when dividing the wild reindeer into regional populations. We further identified nine to ten alleles in each of the two pooled microsatellite sets, and approximately six alleles in each regional population, noting high diversity in all populations (Table 1). As many as 17 and 18 private alleles (approximately 20% of the total number of alleles) were identified in the pooled domestic and wild populations, respectively. As the sample size of wild reindeer is half the size of domestic reindeer, we can assume that there is a higher number of private alleles in the wild populations, implying higher levels of introgression from wild to the domestic genepool than visa‐versa.

**Table 2:** Pairwise genetic differences (*F*_ST_) based on 13 reindeer specific microsatellite loci, among regional populations of domestic (D) and wild (W) herds of reindeer across northeastern Zabaĭkal'e. Population numbers are coded to the abbreviated list in the first column

Code	Population	1	2	3	4	5	6	7
1	Nomama (D)							
2	Lake Nichatka (D)	0.202*						
3	Chapo‐Ologo (D)	0.224*	0.133**					
4	Tia͡nia͡ (D)	0.074^ns^	0.008^ns^	0.062*				
5	Nomama (W)	0.213***	0.162***	0.114***	0.127***			
6	Lake Nichatka (W)	0.407***	0.244***	0.187**	0.200***	0.165**		
7	Tia͡nia͡ (W)	0.426**	0.217**	0.203**	0.163**	0.199**	0.223**	
8	Chapo‐Ologo (W)	0.381***	0.197*	0.175*	0.154*	0.118*	0.111^ns^	0.095^ns^

Significance levels given as ^ns^ *p* > .05, *.01 < *p* < .05, **.001 < *p *< .01, **^*^ *p* < .001.

**Table 3:** Pairwise genetic differences (*F*_ST_) in the mitochondrial CR among regional populations of wild (W) and domestic (D) reindeer across northeastern Zabaĭkal'e. Population numbers are coded to the abbreviated list in the first column

Code	Population	1	2	3	4	5	6	7
1	Nomama (D)							
2	Lake Nichatka (D)	0.027^ns^						
3	Chapo‐Ologo (D)	0.066*	0.000^ns^					
4	Tia͡nia͡ (D)	0.057^ns^	0.000^ns^	0.008^ns^				
5	Nomama (W)	0.000^ns^	0.004^ns^	0.028*	0.017^ns^			
6	Lake Nichatka (W)	0.194***	0.151***	0.166***	0.145***	0.118***		
7	Tia͡nia͡ (W)	0.109*	0.047^ns^	0.041^ns^	0.059^ns^	0.042^ns^	0.000^ns^	
8	Chapo‐Ologo (W)	0.000^ns^	0.000^ns^	0.000^ns^	0.038^ns^	0.000^ns^	0.154*	0.017^ns^

Significant levels given as ^ns^ *p* > .05, *.01 < *p* < .05, **.001 < *p *< .01, ***p < .001.

An AMOVA analysis was run on the entire dataset without imposing any hierarchical structure. It showed that most of the genetic variation is found within populations, rather than among populations, in both datasets (Table 4). When grouping the eight populations into two types (wild and domestic), we found that 2.89% (*p* = .028) of the variance can be attributed to differences between wild and domestic, while 93.60% (*p* < .001) of the variation is found within populations in the microsatellite data. A similar result was evident for the mtDNA data where 4.43% (*p* = .67, not significant) of the variance is found between wild and domestic, while 83% (*p* < .001) is found within populations.

**Table 4:** Results from Analysis of Molecular Variance (AMOVA) from the microsatellite and the mtDNA data. An AMOVA was run on the entire dataset without imposing a hierarchical structure, and by dividing the data into two groups (domestic and wild)

	Microsatellites		MtDNA	
	*F*‐statistic	% of variation	*F*‐statistic	% of variation
No groups: all samples				
Among populations	*F*_ST_: 0.051***	5.11	*F*_ST_: 0.153***	15.28
Within populations		94.89		84.72
Two groups: domestic–wild				
Among groups	*F*_CT_: 0.029*	2.89	*F*_CT_: 0.067^ns^	4.42
Among populations within groups	*F*_SC_: 0.036***	3.52	*F*_SC_: 0.132***	12.57
Within populations	*F*_ST_: 0.064***	93.60	*F*_ST_: 0.170***	83.00

Significance levels given as ^ns^ *p* > .05, *.01 < *p* < .05, **.001 < *p *< .01, **^*^ *p* < .001.

**Figure 3:**
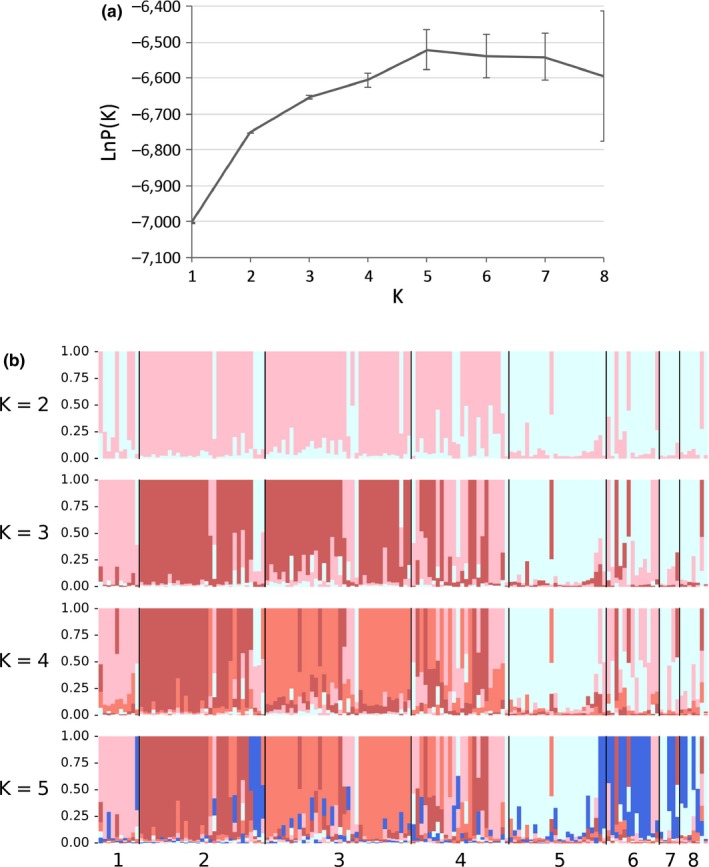
Bayesian clustering analyses of wild and domestic reindeer in northeastern Zabaĭkal'e. (a) Mean likelihood LnP (*K*) over 10 runs dividing the entire dataset into *K* = 1–8 populations, (b) Individual assignment of individual reindeer to each cluster at *K* = 2–5. Populations are coded as in Tables 1 through 3

We analyzed the STRUCTURE results from *K* = 1 to *K* = 5 to assess the mean likelihood of there being separate populations within the dataset. The analysis suggested an apparent main structure at *K* = 2 (Figure 3a), which can be interpreted as a division between wild and domestic herds. The population structure at *K* = 3 is characterized by a separation of most domestic Nomama reindeer from the other domestic herds, while at *K* = 4 it appears that a third domestic population begins to cluster itself around the Lake Nichatka population (Figure 3b). At *K* = 5, although with a greater variety in possible clusters, there is some evidence that the regional population of wild reindeer at Nomama distinguishes itself from other wild populations.

All five clusters are dominated (70%–80%) by individuals from one of the eight populations (Table 5). Except for wild and domestic reindeer at Ti͡ani͡a, all populations had most of its membership belonging to one of the clusters. It is significant that nearly 80% of the domestic reindeer at Nomama presented traits from the domestic population at Chapo‐Ologo. The second STRUCTURE analysis, which was directed only at two inferred populations (wild and domestic), suggests that the Nomama and Ti͡ani͡a domestic herds have relatively high proportion of individuals with mixed ancestry (Table 6). The proportion of individuals with *q* > 0.8 was generally higher among the wild populations than among the domestic populations, implying that the main direction of gene flow has been from the wild to the domestic genepool.

**Table 5:** Proportion of memberships in Bayesian clustering of each pre‐defined Zabaĭkal'e reindeer population in each of the five clusters

	Population	1	2	3	4	5
1	Nomama (D)	0.029	0.029	0.781	0.108	0.053
2	Lake Nichatka (D)	0.119	0.027	0.055	0.111	0.688
3	Chapo‐Ologo (D)	0.709	0.033	0.092	0.057	0.100
4	Tia͡nia͡ (D)	0.185	0.063	0.423	0.080	0.248
5	Nomama (W)	0.054	0.823	0.031	0.064	0.028
6	Lake Nichatka (W)	0.036	0.067	0.057	0.715	0.125
7	Tia͡nia͡ (W)	0.043	0.333	0.272	0.328	0.024
8	Sakukan (W)	0.017	0.604	0.023	0.347	0.009

**Table 6:** Proportion of memberships in Bayesian clustering of each predefined Zabaĭkal'e reindeer population to the domestic or wild gene pool. The assignments are given at three levels of posterior probabilities (*q*) assumed to reflect individuals characterized by ancestry to the correct gene pool (*q *>* *0.8), with mixed ancestry (0.2 < *q *<* *0.8), and ancestry to the alternative gene pool (*q *<* *0.2)

	Population	*N*	Gene pool	*q *>* *0.8	0.2 < *q *<* *0.8	*q *<* *0.2
1	Nomama (D)	10	Domestic	0.30	0.20	0.50
2	Lake Nichatka (D)	31	Domestic	0.88	0.03	0.13
3	Chapo‐Ologo (D)	37	Domestic	0.84	0.11	0.05
4	Ti͡ani͡a (D)	23	Domestic	0.43	0.43	0.13
5	Nomama (W)	24	Wild	0.92	0.08	0.00
6	Lake Nichatka (W)	13	Wild	0.61	0.31	0.08
7	Ti͡ani͡a (W)	7	Wild	0.86	0.14	0.00
8	Sakukan (W)	5	Wild	1.00	0.00	0.00

**Figure 4:**
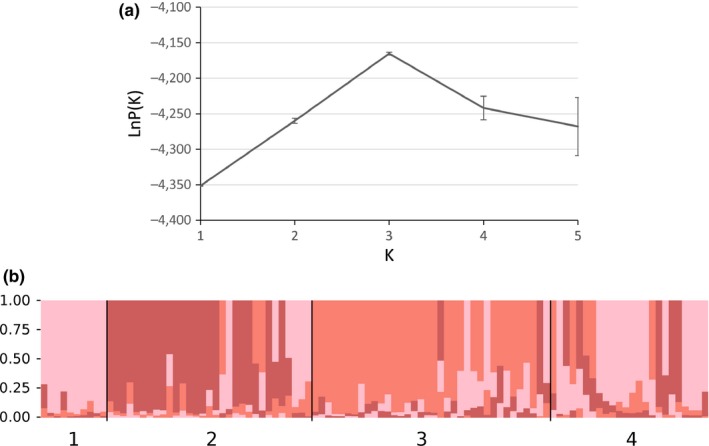
Bayesian clustering analyses for northeastern Zabaĭkal'e including only the domestic animals. (a) Mean likelihood LnP (*K*) over 10 runs dividing the entire dataset into *K* = 1–5 populations, (b) Individual assignment of each individual reindeer to each cluster at *K* = 3 Populations are coded as in Tables 1 through 3

To further test the genetic structure among the domestic reindeer, we re‐ran the STRUCTURE analysis with the 101 domestic samples alone. This analysis supported a separation into three domestic genetic clusters (Figure 4a) with each of Nomama, Lake Nichatka, and Chapo‐Ologo regional herds characterizing the three clusters. The Tiana population appears to be made up of a mixture of all three groups (Figure 4b).

### 3.2 Traditional Evenki herding practices for controlling introgression

**Figure 5:**
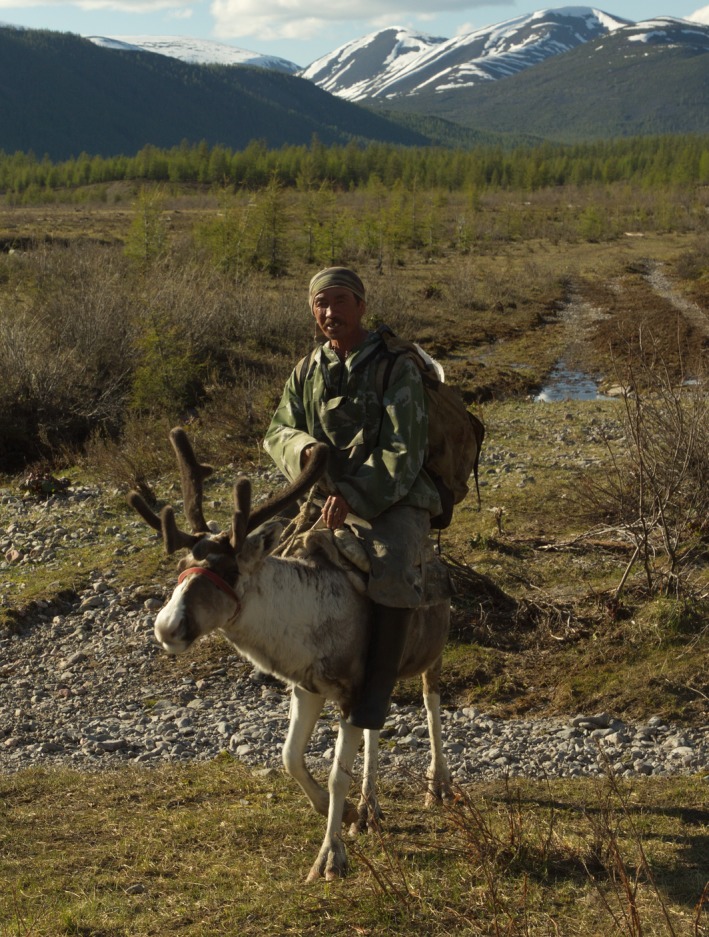
Aleksandr Gabyshev and his trained domestic deer Mal'chik, Amudisy Lakes, Zabaĭkal Kraĭ

Zabaĭkal Evenkis traditionally hold reindeer in small local herds scattered across the region ranging in size from a dozen to two hundred head by extended families of four to twelve individuals. The herd structure varies by the needs of each family and by season, but approximately up to 40% of the herd might be breeding females. The balance would be nonbreeding juveniles or calves, with a handful of males kept as breeding bulls and another dozen males castrated and kept for use as transport reindeer (Figure 5). They generally keep their local herds within the confines of one watershed and are typically not moved more than 50 km over the course of the year. According to our own ethnographic survey, every 5 or 10 years, a herd might be split and then relocated into a neighboring valley forming a new local population. Individual domestic reindeer from one to twenty head, typically bulls, might occasionally be sold and exchanged across regions over a distance of 500 km or more. Overviews of Zabaĭkal Evenki husbandry are available in Orlov (1858); Shirokogoroff (1929), Fondahl (1989) and Anderson (1991); Anderson et al. (2014).

The highly trained domestic reindeer are mainly used for milking and saddling (for transport) and are rarely slaughtered for food (Vasilevich, 1964; Shnirel'man, 1977). This restriction does not apply to the *bai͡unchikar*. The local herdsmen therefore seek to keep their camps near groups of wild forest reindeer, which are an important source of food and skins for clothing and equipment. The wild populations are described as scattered, small, localized groups of ten to twenty animals with a range that intersects with the domestic populations. They do not migrate more than 30 km over the course of a year and alternate their pastures between high alpine meadows and taiga meadows in between mountain ridges (Vodop'i͡anov, 1970a; Ovdin & Ovdin, 2007). An important detail in the interrelationships between these two populations is that the breeding cycle of each is offset. In this region, wild *Rangifer* drop their calves in May 2–3 weeks later than domestic *Rangifer*.

In the local Evenki dialect, wild *Rangifer* (*sakzhoĭ*) and domestic *Rangifer* (*oron*) are marked semantically as different types. The local practice of deliberately interbreeding domestic cows with wild bulls to produce *bai͡unchikar* is noted in the literature but has never been described in detail. The first European to mention the practice was Leopol'd Shrenk (1883) who travelled in the region in the 1850s. One clue to the possible ancient nature of the practice is the fact that all the terminology is in Evenki and not in Russian (or Latin). In our interviews, we documented an “ideal” breeding procedure as practiced in post‐Soviet conditions, which might differ from how this practice was done in Soviet or Imperial times. It was explained that during the autumn rut, the majority of the domestic female herd is sequestered to prevent them from interbreeding. In the case of a small herd, they might be kept overnight in a specially built corral. In the case of a larger herd, they would be herded into an easily monitored valley. In both cases, herdsmen would keep shifts night and day to monitor the herd. However, one or two breeding females—that is, much less than 10% of the female domestic population—might be tethered for cross‐breeding with a wild male. The wild male is not selected as such. He presents himself opportunistically. The interbreeding happens under close observation of the herders. The wild male is often shot after mating, so that he will not harm the female—or, worse, fission‐off a group of domestic cows under his protection by fighting off the other domestic breeding males with his superior s**i**ze and antlers. The act of shooting the wild male is a pure management decision as neither the autumn meat nor the thick hide of the male in rut can be used in subsistence.

The mixed *bai͡unchikan* calves are closely monitored as they grow. The herders watch for a set of behavioral signs that suggest if they are compatible with the rest of the herd. The herders literally describe this as watching to see if the “domestic blood predominates.” There are many qualities that would please a herder such as gregariousness, a lack of aggression, a calm dispossession. Phenotypical qualities are also observed such as the length of the legs, the gait of the calf, and the strength and resilience of the animal. A female *bai͡unchikan* would be monitored to see whether there was a risk she would drop her calves too late in the spring for the calves to be able to outrun predators and therefore survive. The *bai͡unchikan* calves are not separated but are kept with the entire herd, which itself moves through periods of being free‐ranging and free‐foraging to being kept under constant watch, or even being enclosed, depending on the season. If any comment is made about the mixed‐blood calves, it is that they often resist walking together with the herd as a whole that they keep to the edge of the forest and become unruly when enclosed. It is the process of observation and selection which controls genetic introgression. Calves, which present themselves of “wild,” may be allowed to survive one or 2 years as juveniles but would be culled before they reach breeding age. It would be fair to say that the majority of the *bai͡unchikar* in normal economic conditions are not allowed to breed. Female *baikunchar* might be slaughtered for meat before they reach reproductive age. Male crossbreeds may be slaughtered or might be prevented from breeding by castration or by trimming their antlers to prevent them from successfully competing with other males. Some male *bai͡unchikan* castrates are trained to harness to be cargo‐carrying reindeer. Those few mixed breeds allowed to interbreed would be monitored year by year and could always be castrated or culled over the next breeding season. Their calves in the next generation would also be scrutinized. In our field research, the *bai͡unchikar* presented to us were primarily calves. The two adults were castrated males, one of which was “one‐quarter wild.” None of the declared mixed‐breeds at the time of our research could lead to genetic introgression.

The degree to which introgression proceeds depends on local conditions. In our field research, the two adult castrates were born in 2005, one from a male reindeer who was himself a *bai͡unchikan* likely born around 2000. The same dates would apply to one *bai͡unchikan* adult female sampled at Nichatka. This year was a time of severe economic crisis in the region. It was economically impossible hire mechanized transport to bring breeding bulls in from afar, forcing the local herders to recruit the wild population to ensure that all of their cows were covered. In a dire situation like this, one would assume that a higher proportion of the interbred calves would be allowed to survive. This precise situation presented itself before us at Nomama. The previous autumn a wild bull was permitted to cover some of the cows. When bears killed the last breeding domestic male that summer, the herders were making ready to interbreed the remaining domestic females with another wild bull. However, economic opportunities can also encourage interbreeding. There is one published estimate that in 1970 the Soviet state farms of Zabaĭkal'e held between 750 and 800 “hybrid” reindeer out of a total domestic population of 25,000 (Vodop'i͡anov, 1970b). That article makes clear that the peculiar economic conditions of Soviet state socialism—the use of head‐counts and calf‐weights to monitor productivity—encouraged herders to wildly increase the size of the herds through the use of interbreeding. The article also makes clear that the calves are rarely kept beyond a year and a half of age. Finally, all herders will admit candidly that some *bai͡unchikar* are born spontaneously when a small group of domestic females is covered by a wild male unintentionally. The same strict observational rules would apply to those happenstance wild/tame calves.

## 4 DISCUSSION

Our results suggest that the reindeer herders in northeastern Zabaĭkal'e have developed herding techniques that maintain the genetic integrity of coexisting and overlapping populations of wild and domestic reindeer, even while intentional interbreeding them. This implies that the marked genetic differentiation between wild and domestic reindeer previously reported for the Lake Nichatka population (Røed, 2008) reflects a general pattern applicable for other herds across the region as well.

From our genetic analyses, the AMOVA shows that the majority of genetic variation is found within the populations, not between groups, more specifically between wild and domestic. Nevertheless, pairwise genetic distances among all populations reveal relatively high levels of differentiation, especially in the microsatellite data. Further, by pooling the wild and domestic populations, we found significant differentiation—which is also evident from the relatively high number of private alleles found in both wild and domestic pooled populations. A separation between wild and domestic is further supported by the STRUCTURE analysis showing a main structure of two clusters, separating between wild and domestic, although some level of admixture is evident. However, little admixture was shown from the network analysis based on the mitochondrial control region, where only two of a total of 36 haplotypes are shared between wild and domestic reindeer. Significant genetic differences between wild and domestic reindeer have also been reported from Alaska (Cronin, 2003; Mager, 2013; Colson, 2014), Norway (Røed, 2014) and Greenland (Jepsen, 2002). In Norway, wild and domestic herds have been present for several centuries and are usually kept separated in different mountain areas, with enhanced migration barriers due to increasing infrastructure (Røed, 2014). Therefore, classic spatial segregation likely explains the genetic differentiation (Slatkin, 1987). Alaska and Greenland have a relatively recent history of coexistence of wild and domestic herds, which also have different geographic origins. Domestic reindeer were introduced to Alaska from Russia in 1890s (Ellanna, 2005), and to Greenland from Norway in 1952 (Jepsen, 2002). Differences in ancestry, and presumably associated local/regional adaptability, likely have contributed to reproductive isolation, as indicated by their distinct morphological and behavioral differences (Jepsen, 2002; Mager, 2013). For Zabaĭkal'e, the situation is entirely different. Wild and domestic reindeer have the same ancestry, have coexisted for a long time, and are geographically sympatric. The genetic isolation observed between the wild and domestic populations appears rest solely on very effective cultural practices; on how the indigenous herders tightly control breeding between wild and domestic individuals, and monitor the interbreed individuals within the herd. These practices have made it possible for both populations to coexist, selectively interbred, but leaving little trace of long‐term genetic mixture between them. The local herders do not use genetic sampling to guide their practice but rather follow the pedigrees of specific individual animals, often for up to three generations. Their strict monitoring of desirable behavioral qualities in the domestic herd has the latent effect of maintaining the genetic integrity of both wild and domestic populations.

This behavioral factor in selection addresses the recent hypothesis of the existence of “islands of domestication” within the genome wherein selection for desirable behavioral traits allows breeders to maintain domestic behavior within an interbred population (Frantz, 2015). Our results suggest that behavioral selection may indeed be important at the level of individual selection, but there is no evidence in this study suggesting that it can lead to hybridizing an entire population. At the level of single individual cases in each herd, behavior selection is used to permit a small number of hybrid bloodlines into a breeding population. Hence, our data are closer to the model of Warmuth (2012) of the gradual improvement of Eurasian horse herds through the controlled introgression of wild genes along the male line.

Another prominent study of the genetics of animal domestication by Larson and Burger (2013) suggests that early herders may have moved from one place to another with a small number of domestic individuals and then deliberately augmented their herds by continuous admixture with wild individuals in their new place of residence. Their study models the process with archeological samples from pig and wild boar. While elements of our study suggest the use of this technique to pioneer *Rangifer* husbandry—for example, in the case of the Nomama regional population (a substantial portion of which was translocated from Chapo‐Ologo)—our study as whole suggests the reverse: that the genetic integrity of both the wild and domestic populations is maintained. This could be explained by the fact that domestic *Rangifer* are not held in the same way as pigs. It is not sufficient that they tolerate humans and forage independently close‐to‐hand. Reindeer are held in a highly nuanced state of training where they can be harnessed and ridden—a behavioral quality unlikely to be expected of a pig.

The notably low CR haplotype sharing between wild and domestic reindeer, indicate very low levels of female mediated gene flow. This is consistent with female philopatry and male‐biased dispersal commonly seen among most mammal species (Greenwood, 1980; Pusey, 1987; Goudet, 2002). This sex‐biased structure has also been documented for wild *Rangifer* in Alaska (Roffler, 2012). In northeastern Zabaĭkal'e, however, this appears to be greatly aggravated by the strongly sex‐biased and tightly controlled breeding practice. Our ethnographic work suggests that this significant sex‐bias may have been further augmented by the preference for exchanging or importing male domestic reindeer to improve a herd. These small taiga herds are often assembled through kinship. Thus, particular reindeer might be gifted to one family or another when their paths cross in the forest. In our work on reindeer biographies, we discovered that male reindeer in particular might be exchanged several times over between friends and relatives in different locations across hundreds of kilometers with the animal spending 1 or 2 years in various camps (Davydov, 2014). Female animals, on the other hand, are more likely to be kept within the family. Female reindeer would be more likely to be translocated when a herd would grow beyond a certain threshold and relocated to a new valley. This was the case for the local population at Amudisy (Chapo‐Ologo region) which had been fissioned twice as the herd approached 300 head. There are published ethnographic accounts of female reindeer being given in exchange as bride price in the 19th Century (Vasilevich, 1969). Further, female reindeer might be exchanged within large transfers of twenty to fifty head designed to augment a herd, such as the oral accounts of the building of the regional population at Ti͡ani͡a. Our analysis of proportional membership in Table 5 singles out Ti͡ani͡a as a population presenting no strong affinity to any one of the selected populations.

This study was originally designed to test the hypothesis that careful husbandry could successfully keep wild and domestic populations genetically separated despite living in close proximity and despite deliberate attempts to let certain female reindeer to interbreed. The genetic results indicate this is indeed the case. Our results further suggest that it is possible to distinguish local domestic clusters, which may serve as signatures of particular breeding traditions. This finding may be important to bolster local claims to the pedigree status of their herds which could give them access to Russian state funding to preserve pedigree types (Zabrodin & Borozdin, 1989).

This study suggests that it is possible to distinguish regional domestic herds genetically despite complex social histories of collectivization and privatization. The status of the Nomama population is a particularly interesting case in point. Despite significant influxes of reindeer purchased from neighboring regions, and Chapo‐Ologo in particular, the Nomama population continues to distinguish itself from other groups by its low level of genetic diversity within the mtDNA and a strongly different microsatellite signature. The mtDNA signature supports an interpretation that there may have been a founding by a small number of females—perhaps those that oral accounts suggested were lassoed from the free‐ranging feral remnants of the defunct state farm. Their distinct microsatellite signature suggests that the founding population may have been augmented by hybridization with local feral reindeer. Further, the proportion population membership in Table 6 suggests a high probability (50%) that the domestic population is best described as wild. In the older cultural–historical literature, the North Baikal region, where Nomama is located, corresponds to a distinctly different tradition of reindeer herding technique from all other groups (Vasilevich, 1964). In this case, decollectivation and the restoration of family‐based reindeer husbandry between 1976 and 2012 may have produced a small‐scale case study of origin of local reindeer husbandry within a distinct regional population of wild and feral *Rangifer* stressing the importance of strict selection and introgression along the male line.

Up until now, the classic literature on the origin of reindeer husbandry and of reindeer pedigrees has relied upon the classification of saddling and harnessing technologies as a proxy for grouping together distinct indigenous pastoral traditions. The reindeer bodies themselves be they wild or tame were assumed to be standard. This study suggests that in this region of southeastern Siberia, perhaps one of several “hearths” of reindeer husbandry, wild and domestic types are understood to be phenotypically and behaviorally different by local herders, and that this difference extends to the distinct genetic signatures in each regional population. Unlike in other models of the origin of animal husbandry, this study suggests that a respect for difference between the populations leads to a breeding strategy which enhances the genetic diversity of the species as a whole.

## CONFLICT OF INTEREST

None declared.

## DATA ACCESSIBILITY

Genetic mtDNA data generated for this study is available on GenBank. Reference numbers are in Supplementary Table S1.

## Appendix 1

### 1.1

Official government statistics for domestic and wild *Rangifer* for the four districts making up northeastern Zabaĭkal'e. Figures for domestic reindeer are from the Federal State Statistical Service of the Russian Federation (Federal'nai͡a Sluzhba Statistiki, 2016). Figures for wild reindeer are estimated from local hunting ranger reports and are unpublished (Pavlov, 2016)

**Table:**

Region/District	Domestic *Rangifer* (year)	Wild *Rangifer* (year)
Irkutsk oblast’	–	14,111 (2016)
Bodaibo district	357 (2015)	–
Respublika Buri͡atii͡a	–	4,471 (2016)
Severobaikal district	382 (2015)	–
Zabaĭkal Krai	–	4,810 (2016)
Kalar district	273 (2008)	–
Respublika Sakha‐Iakutii͡a		Not available
Olekma district	5,880 (2015)	–

## Appendix 2 Full list of project collaborators.

### 2.1

This was a collaborative project with several indigenous communities in Southeastern Siberia. We greatly appreciate the information shared with us by the leaders of several Evenki communal organizations (*obshchiny*) ‘Tiania’ ‐ Arsentii Nikolaev, ‘Uluki’ ‐ Iurii Chernoev, ‘Oron’ ‐ Aleksei Ganiugin, ‘Tora’ – Iurii Malchakitov. Without their permission to work with their herds, and their help with transport and logistics, we could not have done the fieldwork. We are further grateful to the Evenki reindeer herders and hunters Georgii Lekarev, Pavel Atolainen, Pavel Chernoev, Vladimir Aeul'ev, Evgenii Trynkin, Aleksandr Gabyshev, Gennadii Il'inov, Mikhail Kuz'min, Grigorii Kurbaltunov, Grigorii Nikolaev, Philip Gabyshev, Artur Kul'bertinov, Semen Nektegaev, Anton Pavlov, Konstantin Alekseev, and Sergei Nagovitsyn. They helped us to collect genetic samples and shared with us the history of their herds. We thank Oleg Ganiugin and Aleksandr Kharitonov for their help in gathering wild reindeer genetic samples, and Viktor Ganiugin and Gennadii Kuz'min for sharing their knowledge of Evenki history.

We are also grateful to Lukasz Mikolajczyk for his expert help with the figures.
