# Supplementary material for: Maintaining genetic integrity of coexisting wild and domestic populations: Genetic differentiation between wild and domestic Rangifer with long traditions of intentional interbreeding
Source: Ecol Evol. 2017 Jul 26;7(17):6790–802. doi: 10.1002/ece3.3230 (PMC5587498; doi:10.1002/ece3.3230)
Supplement: Supplementary file 1 [file ECE3-7-6790-s001.docx]

**SUPPORTING INFORMATION**

Maintaining genetic integrity of co-existing wild and domestic populations: Genetic differentiation between wild and domestic *Rangifer* with long traditions of intentional interbreeding

**Authors:** David G. Anderson, Kjersti S. Kvie Vladimir N. Davydov, Knut H. Røed

Table **S1** Details on data set.

| nDNA-label | NVH Lab Label | Type | Sex | Age | Province or Republic | County | Sampling Location | Georeference | GenBank Accession Number |
| --- | --- | --- | --- | --- | --- | --- | --- | --- | --- |
| NoD5003 | Re5003 | D | M | 2 months | Buri͡atii͡a | Severobaĭkalsk | Nomama | 56.305°N 110.344°E | MF039370 |
| NoD5004 | Re5004 | D | F | 3 months | Buri͡atii͡a | Severobaĭkalsk | Nomama | 56.305°N 110.344°E | MF039371 |
| NoD5006 | Re5006 | D | M | 2 years | Buri͡atii͡a | Severobaĭkalsk | Nomama | 56.305°N 110.344°E | MF039373 |
| NoD5007 | Re5007 | D | F | 7-8 years | Buri͡atii͡a | Severobaĭkalsk | Nomama | 56.305°N 110.344°E | MF039374 |
| NoD5008 | Re5008 | D | F | 4 years | Buri͡atii͡a | Severobaĭkalsk | Nomama | 56.305°N 110.344°E | MF039375 |
| NoD5009 | Re5009 | D | M | 4 years | Buri͡atii͡a | Severobaĭkalsk | Nomama | 56.305°N 110.344°E | MF039376 |
| NoD5012 | Re5012 | D | M | 4-5 years | Buri͡atii͡a | Severobaĭkalsk | Nomama | 56.305°N 110.344°E | MF039377 |
| NoD5507 | Re5507 | D | M |  | Buri͡atii͡a | Severobaĭkalsk | Chai͡a River | 56.583°N 110.25°E | MF039378 |
| NoH5005 | Re5005 | D | F | 2 months | Buri͡atii͡a | Severobaĭkalsk | Nomama | 56.305°N 110.344°E | MF039372 |
| NoH5001 | Re5001 | D | M | 2 months | Buri͡atii͡a | Severobaĭkalsk | Nomama | 56.305°N 110.344°E | MF039369 |
| NiD2925 | Re2925 | D | F |  | Chita oblast' | Kalar | Lake Nichatka | 57.766°N 117.65°E | EU653593 |
| NiD2926 | Re2926 | D | F |  | Chita oblast' | Kalar | Lake Nichatka | 57.766°N 117.65°E | EU653605 |
| NiD2927 | Re2927 | D | F |  | Chita oblast' | Kalar | Lake Nichatka | 57.766°N 117.65°E | EU653596 |
| NiD2928 | Re2928 | D | F |  | Chita oblast' | Kalar | Lake Nichatka | 57.766°N 117.65°E | EU653594 |
| NiD2929 | Re2929 | D | F |  | Chita oblast' | Kalar | Lake Nichatka | 57.766°N 117.65°E | EU653597 |
| NiD2930 | Re2930 | D | F |  | Chita oblast' | Kalar | Lake Nichatka | 57.766°N 117.65°E | EU653597 |
| NiD2931 | Re2931 | D | F |  | Chita oblast' | Kalar | Lake Nichatka | 57.766°N 117.65°E | EU653604 |
| NiD2932 | Re2932 | D | F |  | Chita oblast' | Kalar | Lake Nichatka | 57.766°N 117.65°E | EU653599 |
| NiD2933 | Re2933 | D | F |  | Chita oblast' | Kalar | Lake Nichatka | 57.766°N 117.65°E |  |
| NiD2934 | Re2934 | D | M |  | Chita oblast' | Kalar | Lake Nichatka | 57.766°N 117.65°E |  |
| NiD2935 | Re2935 | D | M |  | Chita oblast' | Kalar | Lake Nichatka | 57.766°N 117.65°E |  |
| NiD2936 | Re2936 | D | M |  | Chita oblast' | Kalar | Lake Nichatka | 57.766°N 117.65°E |  |
| NiD2937 | Re2937 | D | M |  | Chita oblast' | Kalar | Lake Nichatka | 57.766°N 117.65°E |  |
| NiD2938 | Re2938 | D | M |  | Chita oblast' | Kalar | Lake Nichatka | 57.766°N 117.65°E | EU653600 |
| NiD2939 | Re2939 | D | M |  | Chita oblast' | Kalar | Lake Nichatka | 57.766°N 117.65°E | EU653601 |
| NiD2940 | Re2940 | D | F |  | Chita oblast' | Kalar | Lake Nichatka | 57.766°N 117.65°E |  |
| NiD2941 | Re2941 | D | F |  | Chita oblast' | Kalar | Lake Nichatka | 57.766°N 117.65°E | EU653602 |
| NiD2942 | Re2942 | D | M |  | Chita oblast' | Kalar | Lake Nichatka | 57.766°N 117.65°E | EU653595 |
| NiD2943 | Re2943 | D | M |  | Chita oblast' | Kalar | Lake Nichatka | 57.766°N 117.65°E | EU653603 |
| NiD2944 | Re2944 | D | F |  | Chita oblast' | Kalar | Lake Nichatka | 57.766°N 117.65°E |  |
| NiD2945 | Re2945 | D | F |  | Chita oblast' | Kalar | Lake Nichatka | 57.766°N 117.65°E |  |
| NiD2946 | Re2946 | D | F |  | Chita oblast' | Kalar | Lake Nichatka | 57.766°N 117.65°E |  |
| NiD2947 | Re2947 | D | F |  | Chita oblast' | Kalar | Lake Nichatka | 57.766°N 117.65°E |  |
| NiD2950 | Re2950 | D | F |  | Chita oblast' | Kalar | Lake Nichatka | 57.766°N 117.65°E | EU653606 |
| NiD2952 | Re2952 | D | U |  | Chita oblast' | Kalar | Lake Nichatka | 57.766°N 117.65°E | EU653609 |
| NiD2953 | Re2953 | D | M |  | Chita oblast' | Kalar | Lake Nichatka | 57.766°N 117.65°E | EU653610 |
| NiD2955 | Re2955 | D | M |  | Chita oblast' | Kalar | Lake Nichatka | 57.766°N 117.65°E |  |
| NiD2956 | Re2956 | D | F | Calf | Chita oblast' | Kalar | Lake Nichatka | 57.766°N 117.65°E | EU653608 |
| NiD2954 | Re2954 | D | M |  | Chita oblast' | Kalar | Lake Nichatka | 57.766°N 117.65°E | EU653607 |
| NiD2958 | Re2958 | D | M | Calf | Chita oblast' | Kalar | Lake Nichatka | 57.766°N 117.65°E |  |
| NiD2959 | Re2959 | D | F |  | Chita oblast' | Kalar | Lake Nichatka | 57.766°N 117.65°E |  |
| ChD5904 | Re5904 | D | M | 1 year | Zabaĭkal Kraĭ | Kalar | Verkhniĭ Sakukan | 56.742°N 117.97°E | MF039392 |
| ChD5905 | Re5905 | D | F | 1 year | Zabaĭkal Kraĭ | Kalar | Verkhniĭ Sakukan | 56.742°N 117.97°E | MF039393 |
| ChD5906 | Re5906 | D | M | 1 year | Zabaĭkal Kraĭ | Kalar | Verkhniĭ Sakukan | 56.742°N 117.97°E | MF039394 |
| ChD5908 | Re5908 | D | M | 1 year | Zabaĭkal Kraĭ | Kalar | Verkhniĭ Sakukan | 56.742°N 117.97°E | MF039396 |
| ChD5909 | Re5909 | D | F | 1 year | Zabaĭkal Kraĭ | Kalar | Verkhniĭ Sakukan | 56.742°N 117.97°E | MF039397 |
| ChD5910 | Re5910 | D | F | 1 year | Zabaĭkal Kraĭ | Kalar | Verkhniĭ Sakukan | 56.742°N 117.97°E | MF039398 |
| ChD5911 | Re5911 | D | F | 2 years | Zabaĭkal Kraĭ | Kalar | Verkhniĭ Sakukan | 56.742°N 117.97°E | MF039399 |
| ChD5912 | Re5912 | D | F | 2 years | Zabaĭkal Kraĭ | Kalar | Verkhniĭ Sakukan | 56.742°N 117.97°E | MF039400 |
| ChD5913 | Re5913 | D | F | 2 years | Zabaĭkal Kraĭ | Kalar | Verkhniĭ Sakukan | 56.742°N 117.97°E | MF039401 |
| ChD5915 | Re5915 | D | F | 3 years | Zabaĭkal Kraĭ | Kalar | Verkhniĭ Sakukan | 56.742°N 117.97°E | MF039403 |
| ChD5916 | Re5916 | D | F | 3 years | Zabaĭkal Kraĭ | Kalar | Verkhniĭ Sakukan | 56.742°N 117.97°E | MF039404 |
| ChD5917 | Re5917 | D | F | 3 years | Zabaĭkal Kraĭ | Kalar | Verkhniĭ Sakukan | 56.742°N 117.97°E | MF039405 |
| ChD5918 | Re5918 | D | F | 3 years | Zabaĭkal Kraĭ | Kalar | Verkhniĭ Sakukan | 56.742°N 117.97°E | MF039406 |
| ChD5919 | Re5919 | D | M | 3 years | Zabaĭkal Kraĭ | Kalar | Verkhniĭ Sakukan | 56.742°N 117.97°E | MF039407 |
| ChD5920 | Re5920 | D | M | 2 years | Zabaĭkal Kraĭ | Kalar | Verkhniĭ Sakukan | 56.742°N 117.97°E | MF039408 |
| ChD5921 | Re5921 | D | M | 2 years | Zabaĭkal Kraĭ | Kalar | Verkhniĭ Sakukan | 56.742°N 117.97°E | MF039409 |
| ChD5922 | Re5922 | D | M | 3 years | Zabaĭkal Kraĭ | Kalar | Verkhniĭ Sakukan | 56.742°N 117.97°E | MF039410 |
| ChD5923 | Re5923 | D | M | 13 years | Zabaĭkal Kraĭ | Kalar | Verkhniĭ Sakukan | 56.742°N 117.97°E | MF039411 |
| ChD5925 | Re5925 | D | M | 8 years | Zabaĭkal Kraĭ | Kalar | Verkhniĭ Sakukan | 56.742°N 117.97°E | MF039413 |
| ChD5926 | Re5926 | D | M | 7 years | Zabaĭkal Kraĭ | Kalar | Verkhniĭ Sakukan | 56.742°N 117.97°E | MF039414 |
| ChD5924 | Re5924 | D | M | 9 years | Zabaĭkal Kraĭ | Kalar | Verkhniĭ Sakukan | 56.742°N 117.97°E | MF039412 |
| ChD5914 | Re5914 | D | F | 2 years | Zabaĭkal Kraĭ | Kalar | Verkhniĭ Sakukan | 56.742°N 117.97°E | MF039402 |
| ChD5907 | Re5907 | D | M | 1 year | Zabaĭkal Kraĭ | Kalar | Verkhniĭ Sakukan | 56.742°N 117.97°E | MF039378 |
| ChD5457 | Re5457 | D | F | 1.5 years | Zabaĭkal Kraĭ | Kalar | Amudisy | 56.683°N 119.032°E | MF039379 |
| ChD5458 | Re5458 | D | F | 1.5 years | Zabaĭkal Kraĭ | Kalar | Amudisy | 56.683°N 119.032°E | MF039380 |
| ChD5468 | Re5468 | D | M | 4 years | Zabaĭkal Kraĭ | Kalar | Amudisy | 56.683°N 119.032°E | MF039382 |
| ChD5470 | Re5470 | D | M | 0.5 years | Zabaĭkal Kraĭ | Kalar | Amudisy | 56.683°N 119.032°E | MF039383 |
| ChD5471 | Re5471 | D | M | 4 years | Zabaĭkal Kraĭ | Kalar | Amudisy | 56.683°N 119.032°E | MF039384 |
| ChD5474 | Re5474 | D | M | 4 years | Zabaĭkal Kraĭ | Kalar | Amudisy | 56.683°N 119.032°E | MF039385 |
| ChD5482 | Re5482 | D | M | 1 month | Zabaĭkal Kraĭ | Kalar | Amudisy | 56.683°N 119.032°E | MF039388 |
| ChD5521 | Re5521 | D | F | 2 years | Sakha-Iakutii͡a | Olekma | Olonnokon River | 57.63°N 120.174°E | MF039390 |
| ChD5527 | Re5527 | D | M | 5 years | Sakha-Iakutii͡a | Olekma | Olonnokon River | 57.629°N 120.174°E | MF039391 |
| ChD5518 | Re5518 | D | M | 8 years | Sakha-Iakutii͡a | Olekma | Murkugu bilire | 58.043°N 121.008°E | MF039389 |
| ChD5462 | Re5462 | D | M | 3 years | Zabaĭkal Kraĭ | Kalar | Amudisy | 56.683°N 119.032°E | MF039381 |
| ChH5478 | Re5478 | FW | M | 1 year | Zabaĭkal Kraĭ | Kalar | Amudisy | 56.683°N 119.032°E | MF039386 |
| ChH5479 | Re5479 | FW | F | 2 months | Zabaĭkal Kraĭ | Kalar | Amudisy | 56.683°N 119.032°E | MF039387 |
| TiD5455 | Re5455 | D | M | 7-8 years | Zabaĭkal Kraĭ | Kalar | Amudisy | 56.683°N 119.032°E | MF039415 |
| TiD5459 | Re5459 | D | M | 6 years | Zabaĭkal Kraĭ | Kalar | Amudisy | 56.683°N 119.032°E | MF039416 |
| TiD5460 | Re5460 | D | M | adult | Zabaĭkal Kraĭ | Kalar | Amudisy | 56.683°N 119.032°E | MF039417 |
| TiD5462 | Re5461 | D | M | 7 years | Zabaĭkal Kraĭ | Kalar | Amudisy | 56.683°N 119.032°E | MF039418 |
| TiD5464 | Re5464 | D | M | 4 years | Zabaĭkal Kraĭ | Kalar | Amudisy | 56.683°N 119.032°E | MF039419 |
| TiD5466 | Re5466 | D | M | 8 years | Zabaĭkal Kraĭ | Kalar | Amudisy | 56.683°N 119.032°E | MF039420 |
| TiD5467 | Re5467 | D | F | 1 year | Zabaĭkal Kraĭ | Kalar | Amudisy | 56.683°N 119.032°E | MF039421 |
| TiH5480 | Re5480 | FW | M | 8 year | Zabaĭkal Kraĭ | Kalar | Amudisy | 56.683°N 119.032°E | MF039422 |
| TiD5508 | Re5508 | D | M | 5 years | Sakha-Iakutii͡a | Olekma | Torgo River | 58.435°N 119.595°E | MF039423 |
| TiD5509 | Re5509 | D | M | 3 years | Sakha-Iakutii͡a | Olekma | Usu River | 57.955°N 120.357°E | MF039424 |
| TiD5520 | Re5520 | D | M | 2 years | Sakha-Iakutii͡a | Olekma | Olonnokon River | 57.63°N 120.174°E | MF039434 |
| TiD5522 | Re5522 | D | M | 4 years | Sakha-Iakutii͡a | Olekma | Olonnokon River | 57.63°N 120.174°E | MF039435 |
| TiD5523 | Re5523 | D | M | 6 years | Sakha-Iakutii͡a | Olekma | Olonnokon River | 57.63°N 120.174°E | MF039436 |
| TiD5525 | Re5525 | D | M | 10 years | Sakha-Iakutii͡a | Olekma | Olonnokon River | 57.629°N 120.174°E | MF039437 |
| TiD5526 | Re5526 | D | M | 4 years | Sakha-Iakutii͡a | Olekma | Olonnokon River | 57.629°N 120.174°E | MF039438 |
| TiD5510 | Re5510 | D | F | 10 years | Sakha-Iakutii͡a | Olekma | Ori͡us-Miele River | 57.838°N 121.106°E | MF039425 |
| TiD5512 | Re5512 | D | F | 10 years | Sakha-Iakutii͡a | Olekma | Ori͡us-Miele River | 57.838°N 121.106°E | MF039427 |
| TiD5513 | Re5513 | D | F | 10 years | Sakha-Iakutii͡a | Olekma | Ori͡us-Miele River | 57.838°N 121.106°E | MF039428 |
| TiD5514 | Re5514 | D | F | 3 years | Sakha-Iakutii͡a | Olekma | Ori͡us-Miele River | 57.838°N 121.106°E | MF039429 |
| TiD5515 | Re5515 | D | M | 5 years | Sakha-Iakutii͡a | Olekma | Murkugu bilire | 58.043°N 121.008°E | MF039430 |
| TiD5516 | Re5516 | D | F | 5 years | Sakha-Iakutii͡a | Olekma | Murkugu bilire | 58.043°N 121.008°E | MF039431 |
| TiD5517 | Re5517 | D | M | 7 years | Sakha-Iakutii͡a | Olekma | Murkugu bilire | 58.043°N 121.008°E | MF039432 |
| TiD5519 | Re5519 | D | M | 10 years | Sakha-Iakutii͡a | Olekma | Murkugu bilire | 58.043°N 121.008°E | MF039433 |
| TiH5511 | Re5511 | FW | M | 8 years | Sakha-Iakutii͡a | Olekma | Ori͡us-Miele River | 57.838°N 121.106°E | MF039426 |
| NoW5002 | Re5002 | W | M |  | Buri͡atii͡a | Severobaĭkalsk | Nomama | 56.305°N 110.344°E | MF039458 |
| NoW5483 | Re5483 | W | F |  | Buri͡atii͡a | Severobaĭkalsk | Chai͡a River | 56.583°N 110.25°E | MF039440 |
| NoW5484 | Re5484 | W | F |  | Buri͡atii͡a | Severobaĭkalsk | Chai͡a River | 56.583°N 110.25°E | MF039441 |
| NoW5485 | Re5485 | W | F |  | Buri͡atii͡a | Severobaĭkalsk | Tyi River | 56°N 109.5°E | MF039442 |
| NoW5486 | Re5486 | W | F |  | Buri͡atii͡a | Severobaĭkalsk | Tyi River | 56°N 109.5°E | MF039443 |
| NoW5487 | Re5487 | W | U |  | Buri͡atii͡a | Severobaĭkalsk | Uian | 56°N 110°E | MF039444 |
| NoW5488 | Re5488 | W | U |  | Buri͡atii͡a | Severobaĭkalsk | Akuli | 56°N 110°E | MF039445 |
| NoW5489 | Re5489 | W | U |  | Buri͡atii͡a | Severobaĭkalsk | Nomama | 56.323°N 110.223°E | MF039446 |
| NoW5490 | Re5490 | W | U |  | Buri͡atii͡a | Severobaĭkalsk | Nomama | 56.323°N 110.223°E | MF039447 |
| NoW5491 | Re5491 | W | U |  | Buri͡atii͡a | Severobaĭkalsk | Nomama | 56.323°N 110.223°E | MF039448 |
| NoW5492 | Re5492 | W | U |  | Buri͡atii͡a | Severobaĭkalsk | Nomama | 56.323°N 110.223°E | MF039449 |
| NoW5493 | Re5493 | W | U |  | Buri͡atii͡a | Severobaĭkalsk | Nomama | 56.323°N 110.223°E | MF039450 |
| NoW5494 | Re5494 | W | U |  | Buri͡atii͡a | Severobaĭkalsk | Nomama | 56.323°N 110.223°E | MF039451 |
| NoW5495 | Re5495 | W | U |  | Buri͡atii͡a | Severobaĭkalsk | Nomama | 56.323°N 110.223°E | MF039452 |
| NoW5496 | Re5496 | W | U |  | Buri͡atii͡a | Severobaĭkalsk | Chai͡a River | 56.583°N 110.25°E | MF039453 |
| NoW5497 | Re5497 | W | U |  | Buri͡atii͡a | Severobaĭkalsk | Chai͡a River | 56.583°N 110.25°E | MF039454 |
| NoW5498 | Re5498 | W | U |  | Buri͡atii͡a | Severobaĭkalsk | Chai͡a River | 56.583°N 110.25°E | MF039455 |
| NoW5499 | Re5499 | W | U |  | Buri͡atii͡a | Severobaĭkalsk | Chai͡a River | 56.583°N 110.25°E | MF039456 |
| NoW5501 | Re5501 | W | U |  | Buri͡atii͡a | Severobaĭkalsk | Chai͡a River | 56.583°N 110.25°E | MF039457 |
| NoW5502 | Re5502 | W | U |  | Buri͡atii͡a | Severobaĭkalsk | Chai͡a River | 56.583°N 110.25°E | MF039458 |
| NoW5503 | Re5503 | W | U |  | Buri͡atii͡a | Severobaĭkalsk | Chai͡a River | 56.583°N 110.25°E | MF039459 |
| NoW5504 | Re5504 | W | U |  | Buri͡atii͡a | Severobaĭkalsk | Chai͡a River | 56.583°N 110.25°E | MF039460 |
| NoW5505 | Re5505 | W | U |  | Buri͡atii͡a | Severobaĭkalsk | Chai͡a River | 56.583°N 110.25°E | MF039461 |
| NoW5506 | Re5506 | W | U |  | Buri͡atii͡a | Severobaĭkalsk | Chai͡a River | 56.583°N 110.25°E | MF039462 |
| NiW2960 | Re2960 | W | F |  | Chita oblast' | Kalar | Lake Nichatka | 57.766°N 117.65°E | EU653615 |
| NiW2961 | Re2961 | W | M | Adult | Chita oblast' | Kalar | Lake Nichatka | 57.766°N 117.65°E | EU653613 |
| NiW2962 | Re2962 | W | U |  | Chita oblast' | Kalar | Lake Nichatka | 57.766°N 117.65°E |  |
| NiW2963 | Re2963 | W | U |  | Chita oblast' | Kalar | Lake Nichatka | 57.766°N 117.65°E | EU653611 |
| NiW2964 | Re2964 | W | U |  | Chita oblast' | Kalar | Lake Nichatka | 57.766°N 117.65°E | EU653614 |
| NiW2965 | Re2965 | W | U |  | Chita oblast' | Kalar | Lake Nichatka | 57.766°N 117.65°E | EU653616 |
| NiW2966 | Re2966 | W | M |  | Chita oblast' | Kalar | Lake Nichatka | 57.766°N 117.65°E | EU653612 |
| NiW2967 | Re2967 | W | U | Adult | Chita oblast' | Kalar | Lake Nichatka | 57.766°N 117.65°E | EU653617 |
| NiW2968 | Re2968 | W | U |  | Chita oblast' | Kalar | Lake Nichatka | 57.766°N 117.65°E | EU653620 |
| NiW2969 | Re2969 | W | F | Adult | Chita oblast' | Kalar | Lake Nichatka | 57.766°N 117.65°E |  |
| NiW2970 | Re2970 | W | F | Adult | Chita oblast' | Kalar | Lake Nichatka | 57.766°N 117.65°E | EU653618 |
| NiW2971 | Re2971 | W | M |  | Chita oblast' | Kalar | Lake Nichatka | 57.766°N 117.65°E |  |
| NiW2972 | Re2972 | W | F |  | Chita oblast' | Kalar | Lake Nichatka | 57.766°N 117.65°E | EU653619 |
| ChW5475 | Re5475 | W | U |  | Zabaĭkal Kraĭ | Kalar | Amudisy | 56.683°N 119.032°E | MF039463 |
| ChW5476 | Re5476 | W | U |  | Zabaĭkal Kraĭ | Kalar | Amudisy | 56.683°N 119.032°E | MF039464 |
| ChW5477 | Re5477 | W | U |  | Zabaĭkal Kraĭ | Kalar | Amudisy | 56.683°N 119.032°E | MF039465 |
| ChW5481 | Re5481 | W | M | 1.5 years | Zabaĭkal Kraĭ | Kalar | Amudisy | 56.683°N 119.032°E | MF039466 |
| ChW5927 | Re5927 | W | M | 1 year | Zabaĭkal Kraĭ | Kalar | Torochan | 56.683°N 119.032°E | MF039467 |
| TiW5529 | Re5529 | W | M |  | Sakha-Iakutii͡a | Olekma | Usu River | 57.955°N 120.357°E | MF039472 |
| TiW5530 | Re5530 | W | U |  | Sakha-Iakutii͡a | Olekma | Usu River | 57.955°N 120.357°E | MF039473 |
| TiW5531 | Re5531 | W | U |  | Sakha-Iakutii͡a | Olekma | Usu River | 57.955°N 120.357°E | MF039474 |
| TiW5533 | Re5533 | W | M | 12 years | Sakha-Iakutii͡a | Olekma | Ori͡us-Miele River | 57.838°N 121.106°E | MF039468 |
| TiW5534 | Re5534 | W | M | 6 years | Sakha-Iakutii͡a | Olekma | Ori͡us-Miele River | 57.838°N 121.106°E | MF039469 |
| TiW5535 | Re5535 | W | F | 3 years | Sakha-Iakutii͡a | Olekma | Ori͡us-Miele River | 57.838°N 121.106°E | MF039470 |
| TiW5536 | Re5536 | W | F | 2 years | Sakha-Iakutii͡a | Olekma | Ori͡us-Miele River | 57.838°N 121.106°E | MF039471 |
